# Circular RNA VRK1 facilitates pre‐eclampsia progression via sponging miR‐221‐3P to regulate PTEN/Akt

**DOI:** 10.1111/jcmm.16454

**Published:** 2021-03-18

**Authors:** Ziwei Li, Xinyi Zhou, Wenyan Gao, Manni Sun, Haiying Chen, Tao Meng

**Affiliations:** ^1^ Department of Obstetrics The First Affiliated Hospital of China Medical University Shenyang China; ^2^ China Medical University Shenyang China

**Keywords:** circular RNA, competing endogenous RNA, gene expression profiling, MicroRNA, pre‐eclampsia

## Abstract

Pre‐eclampsia (PE) is a worldwide pregnancy‐related disorder. It is mainly characterized by defect migration and invasion of trophoblast cells. Recently, circular RNAs (circRNAs) have been believed to play a vital role in PE. The expression patterns and the biological functions of circRNAs in PE remain elusive. Here, we performed a circRNA microarray to identify putative PE‐related circRNAs. Bioinformatics analyses were used to screen the circRNAs which have potential relationships with pre‐eclampsia, and we identified a novel circRNA (circVRK1) that was up‐regulated in PE placenta tissues. By using HTR‐8/SVneo cells, circVRK1 knockdown significantly enhanced cell migration and invasion abilities, as well as epithelial‐mesenchymal transition (EMT). Mechanistically, we found that circVRK1 and PTEN could function as the ceRNAs to miR‐221‐3p. Overexpression of miR‐221‐3p promoted cell migration, invasion and EMT via regulating PTEN. The cotransfection of miR‐221‐3p inhibitor or PTEN reversed the effect from circVRK1 knockdown. Moreover, the circVRK1/miR‐221‐3p/PTEN axis greatly regulated Akt phosphorylation. In general, circVRK1 suppresses trophoblast cell migration, invasion and EMT, by acting as a ceRNA to miR‐221‐3p to regulate PTEN, and further inhibit PI3K/Akt activation. The purpose of this paper is to open wide insights to investigate the onset of PE and provide new potential therapeutic targets in PE.

## 1 INTRODUCTION

Pre‐eclampsia (PE) refers to a condition associated with pregnancy that presents new‐onset hypertension with or without proteinuria, often starting from the 20th week of gestation.^1^ It is a common obstetric disorder that remains the main contributor to neonatal and maternal mortality and morbidity, targeting 2%‐8% of all pregnancy cases worldwide.^1^, ^2^, ^3^ The precise etiopathogenesis of pre‐eclampsia is believed to be multifactorial.^4^ Accumulating studies have shown that extravillous trophoblasts (EVTs) play important roles in placenta development, their limited migration and invasion abilities can lead to abnormal spiral artery remodelling^5^ and defective trophoblast invasion.^6^ Moreover, epithelial‐to‐mesenchymal transition (EMT) of trophoblast cells is a progression characterized by trophoblast cells transform from epithelial phenotype into mesenchymal phenotype, getting characteristics containing enhanced migration and invasion abilities.^7^ So that trophoblast cells can anchor the placenta into maternal tissues and remodel the maternal spiral arterioles.^8^ Therefore, the poor migration and invasion abilities of trophoblast cells were believed to be essential leading causes of PE. It is of great significance to explore the molecular mechanisms that drive the progression of pre‐eclampsia, which may provide more effective clinical therapies.

Circular RNAs (circRNAs) are a type of endogenous non‐coding RNAs defined by continuous loops closed by covalent binding without 5′ to 3′ polarity and polyadenylated tails.^9^, ^10^ This type of RNA is formed by back‐splicing events, resulting in enhanced stability relative to linear RNA.^11^ In recent years, studies have proved that circRNAs participate in various diseases.^12^, ^13^ Competing endogenous RNA (ceRNA) is one of the functional roles of circRNAs which is involved in the metastasis of the diseases.^14^, ^15^, ^16^ A circRNA functions as a miRNA sponge as it binds to the relevant miRNA and influences the generation of miRNAs that interact with their target mRNAs.^14^, ^17^ However, few studies demonstrated the regulation mechanism of circRNAs in PE. For example, Shen et al^18^ found that hsa_circ_0006772 can inhibit trophoblast cell migration and EMT via the miR‐762/Grhl2 pathway. Down‐regulated hsa_circ_0088227 suppresses trophoblast cell proliferation and invasion by targeting miR‐384.^19^ Here, we believe that circRNAs play critical functional roles in PE.

In this study, we identified a circular RNA (circVRK1) by microarray from the placentas of PE and NP. It termed that circVRK1 was up‐regulated in PE and could function as a ceRNA to PTEN to suppress trophoblast cell migration, invasion and EMT by targeting miR‐221‐3P. The circVRK1/miR‐221‐3p/PTEN also influenced the PI3K/Akt signal pathway. Overall, our study demonstrated that circVRK1 serves as a potential biomarker for the diagnosis of PE, hoping to provides new insights to study the pathogenesis of PE.

## 2 MATERIALS AND METHODS

### 2.1 Samples collection and patient enrolment

A careful selection was done to enroll 74 pregnant women undergoing caesarean surgery at The First Hospital of China Medical University (Department of Obstetrics), Liaoning, China, from October 2017 to September 2020. This study was approved by the Ethics Committee of The First Hospital of China Medical University, and all participants signed informed consent. PE is characterized by diastolic blood pressure (mm Hg ≥ 90) or systolic blood pressure (mm Hg ≥ 140) after 20 weeks of gestation (indicating new‐onset hypertension), with new‐onset proteinuria (≥0.3 g/24 h or ≥2 + examined using a dipstick test for two random specimens). In this study, we included patients that were diagnosed with severe pre‐eclampsia which is defined with PE with severe features according to ACOG (systolic blood pressure of 160 mm Hg or higher, or diastolic blood pressure of 110 mm Hg or higher, any of thrombocytopenia, impaired liver function as indicated by abnormally elevated blood concentrations of liver enzymes and severe persistent right upper quadrant or epigastric pain unresponsive to medication and not accounted for by alternative diagnoses, renal insufficiency, pulmonary oedema, new‐onset headache unresponsive to medication and not accounted for by alternate, visual disturbance),^1^ to avoid the influence of non‐placental factors in mild pre‐eclampsia. We excluded twin pregnancy, gestational diabetes, renal disease, chronic hypertension, acute or chronic hepatitis, hyperthyroidism, and hypothyroidism. The participants were divided by early‐onset PE (PE occurs before 34 gestational ages), late‐onset PE (PE occurs after 34 gestational ages) and natural pregnancy.^20^ The characteristics were provided in Table 1. Tissues from the maternal surface of the placentas were dissected, cleaned thrice with diethylpyrocarbonate‐treated saline to wash out blood after which they were snap‐frozen in liquid nitrogen. These procedures were completed within 15 minutes after the caesarean sections. Then, the tissues were stored at −80°C.

**TABLE 1:** Clinical characteristics of pregnancies in this research

Characteristics	Early‐onset PE (n = 21)	Late‐onset PE (n = 25)	NP (n = 28)
Maternal age (y)	29.86 ± 4.43	30.04 ± 3.23	30.32 ± 4.40
Gestational age (wk)	31.96 ± 1.07	36.40 ± 0.83^a^	38.70 ± 0.77
Body Mass Index (kg/m^2^)	29.85 ± 2.47	31.03 ± 3.42	28.25 ± 3.68
Systolic BP (mm Hg)	172.14 ± 14.92	168.88 ± 12.47	112.25 ± 6.10^b^, ^c^
Diastolic BP (mm Hg)	114.43 ± 13.22	108.04 ± 11.36	72.57 ± 9.85^b^, ^c^
24‐h proteinuria quantification (g/24)	13.12 ± 9.67	6.23 ± 4.85	–
Proteinuria level (mg/dL)	965.11 ± 783.75	456.49 ± 387.20	–
Urine (mL/24 h)	1662.38 ± 772.06	1441.20 ± 378.31	1192.86 ± 225.96
PLT (10^9^/L)	166.05 ± 62.66	182.48 ± 59.64	214.86 ± 48.03^b^, ^c^
AST (U/L)	33.48 ± 23.81	23.76 ± 14.43	15.04 ± 4.06^b^, ^c^
ALT (U/L)	26.57 ± 21.43	18.68 ± 10.81	13.93 ± 4.61^b^, ^c^
Creatinine (mg/dL)	0.877 ± 0.31	0.73 ± 0.22^a^	0.63 ± 0.80^b^, ^c^
Birth weight (g)	1660.48 ± 419.63	2702.40 ± 427.66^a^	3576.43 ± 305.62^b^, ^c^
Birth length (cm)	38.95 ± 3.68	46.96 ± 2.30^a^	50.46 ± 1.32^b^, ^c^
Apgar (1 min)	8.52 ± 1.17	9.40 ± 0.71	10^b^, ^c^

Note

Values are shown as mean ± SD.

^a^ Early‐onset PE group compared with late‐onset PE group.

^b^ Early‐onset PE group compared with NP group.

^c^ Late‐onset PE group compared with NP group, *P * < 0.05.

### 2.2 Total RNA extraction

TRIzol reagent (Invitrogen) was used to extract total tissue RNA from PE and NP specimens following the instructions on the product manual. The purity and concentration of the RNA were evaluated based on OD 260/280 readings performed from a NanoDrop ND‐1000 machine (Thermo Fisher Scientific).

### 2.3 Microarray and analysis of circRNA expression profile

Total RNA was evaluated with a NanoDrop ND‐1000 and processed using RNase R (Epicentre, Inc) for circRNA enrichment and linear RNA removal. This was followed by amplification and transcription of the enriched circRNAs to fluorescent cRNAs. The Arraystar Human circRNA Array V2 (8x15K, Arraystar) was used to hybridize the labelled cRNAs which were incubated at 65°C for 17 hours in an Agilent hybridization oven. After washing, slides were captured with an Agilent Scanner G2505C. The Agilent Feature Extraction software (version 11.0.1.1) was used for data analysis. Data processing and quantile normalization were carried out by the R software limma package. In our study, a *P*‐value of ≤0.05 and a fold change of ≥2 were set as the cut‐off for identifying differentially expressed circRNAs between the PE and NP groups. The *t* test was used to determine statistical significance. The Benjamini‐Hochberg FDR method was applied to determine the false discovery rate to correct the *P*‐values. The human reference genome (hg19) was used to align all differentially expressed circRNAs. Hierarchical clustering was performed using MeV (Multiple Experiment Viewer) to identify circRNAs with vastly altered expression. The R package Bioconductor was utilized to carry out GO enrichment analysis and Kyoto Encyclopedia of Genes and Genomes (KEGG) pathway enrichment analysis for the differentially expressed circRNAs.

### 2.4 Cell culture and transfection

Human extravillous trophoblasts cell line HTR‐8/SVneo cells were generously provided by Dr Charles H. Graham (Queen's University) and cultured in RPMI‐1640 (Gibco) with 10% fetal bovine serum (FBS, Hyclone). Cells were seeded in six‐well plates at 2 × 10^5^ cells/well for 48 hours at 37°C with 5% CO_2_. siRNA (small interfering RNA) of circVRK1 (si‐circVRK1), siRNA negative control (si‐NC), shRNA (short‐hairpin RNA) of circVRK1 (sh‐circVRK1), shRNA negative control (sh‐NC) and overexpression plasmids PTEN (PTEN) were purchased by Syngentech Company, miR‐221‐3p mimic (miR‐221‐3p), miR‐221‐3p negative control, miR‐221‐3p inhibitor and inhibitor negative control were generated by Riobo Company. After seeding, cells were transfected using lipofectamine 3000 according to the manufacture's construction.

### 2.5 Reverse transcription and quantitative real‐time polymerase chain reaction

qPCR was carried out on a ViiA 7 Real‐time PCR System (Applied Biosystems) by standard procedures using the total RNA obtained from NP and PE specimens by TRIzol reagent (Invitrogen). cDNA was processed from the RNA by a reverse transcription reaction using random primers and a Transcriptor First Strand cDNA Synthesis Kit (Roche). The reaction conditions were in the following sequence: 95°C for 10 minutes, 40 cycles of 95°C for 10 seconds, 60°C for 60 seconds, and 95°C for 15 seconds. The relative expression level of circRNAs was calculated using the ΔCt method and after normalization to the expression level of the housekeeping gene GAPDH.

### 2.6 RNase R treatment

The treatment was performed under the manufacturer's instructions. Briefly, total RNA after extracting by Trizol reagent was incubated with 3 U/μg of RNase R (Epicentre Technologies) for 20 minutes at 37°C. Then, the treated RNA was purified by RNA clean kit (Tiangen) according to the guidance.

### 2.7 RNA fluorescence in situ hybridization (FISH)

We used specific probes for circVRK1 (cy3‐labelled) and miR‐221‐3p (FAM‐labelled) in situ hybridizations. Nuclei were stained using 4,6‐diamidino‐2‐phenylindole (DAPI). The procedures were performed following the manufacturer's instructions (Servicebio). Then, the images were captured under a microscope (Nikon Eclpse Ti‐sr Microscope).

### 2.8 Cell migration and invasion assay

Transwell assay was used to explore trophoblast cell migration and invasion abilities. HTR‐8/SVneo cells (1 × 10^5^ cells) were transfected and plated in the upper chambers of the transwell filters (8 μm; Corning Incorporated, Corning) which were precoated with or without 50 μL of Matrigel (BD Biosciences). The lower chambers were placed with RPMI 1640 medium containing 10% FBS. The cells were cultured at 37°C for 48 hours for invasion assay and 24 hours for migration assay. After incubation, the upper chambers were removed with cotton swabs, the lower chambers were fixed with methanol and stained with crystal violet. Images were obtained by a microscope.

Wound assay was performed to detect cell migration. HTR‐8/SVneo cells were seeded in six‐well plates (1 × 10^6^ cells); the single‐cell layer was wound using 200 μL pipette tip and then washed three times with PBS to remove cell debris. RPMI‐1640 without FBS was added to each well and incubate for 48 hours at 37°C with 5% CO_2_. Images of the wound were obtained at 0 and 48 hour at the same scratch position by a microscope.

### 2.9 Immunofluorescence analysis

HTR‐8/SVneo cells were seeded in six‐well plates at 37°C with 5% CO_2_ and then fixed in 4% paraformaldehyde after washed with PBS two times. Cells were incubated with primary antibodies of E‐cadherin and vimentin for 1 hour (Table S1). After corresponding with fluoresce‐labelled secondary antibodies and DAPI for nuclear counterstaining, microscopy was used to catch the image.

### 2.10 Western blot

Total protein was extracted by radioimmunoprecipitation assay lysis buffer (RIPA, Solarbio) and then separated by SDS–PAGE to polyvinylidene difluoride membranes (PVDF, Millipore). After blocking, the PVDF, membranes were incubated overnight at 4°C with primary antibody E‐cadherin, N‐cadherin, vimentin, twist1, snail, ZEB1, ZEB2, PTEN, p‐Akt and Akt (Table S1). The PVDF membranes were incubated with the secondary antibody for 2 hours at room temperature after washing with TBST. The bands were visualized using a standard protocol for electrochemiluminescence (New Cell & Molecular Biotech, Co). GAPDH was used as the internal standard.

### 2.11 RNA pull‐down assay

Biotin‐labelled circ VRK1 probe and control probe (Sangon Biotech) were used for pull‐down assay. In short, the probe was incubated with magnetic beads (Life Technologies) to generate probe‐coated beads and then incubated with the lysed HTR‐8/SVneo cell samples overnight at 37°C. On the next day, after washing, the sample was incubated with lysis buffer and proteinase K buffer. Finally, TRIzol reagent was added into the complex for RNA extraction. qRT‐PCR was performed to test the pull‐down RNAs.

### 2.12 RNA pull‐down sequencing

The enriched miRNAs by RNA pull‐down were taken to construct a library that will be suitable for the Illumina high‐throughput sequencing platform with VAHTS Small RNA Library Prep Kit for Illumina. The miRNAs were, respectively, connected with universal adapters at 3′ and 5′, and then underwent reverse transcription, PCR expansion and magnetic bead purification to finally obtain a stepwise library suitable for the Illumina platform. At the same time, the stability and reproducibility of the library are improved through strict quality control of the library.

### 2.13 Dual‐luciferase reporter assay

A dual‐luciferase reporter assay was performed to determine the interaction of the RNAs. circVRK1 sequence containing the predicted binding site of miR‐221‐3p wild (circVRK1‐WT) or mutant (circVRK1‐Mut) was subcloned into the pmirGLO vector (Promega). 293T cells were seeded in 24‐well plates (5 × 10^5^ cells), and cotransfected with the circVRK1‐WT or circVRK1‐Mut together with miR‐221‐3p mimics or NC. After 48 hours, the luciferase activity was measured and normalized to the Renilla luciferase.

### 2.14 Data analysis

Quantitative data are expressed as means ± SD. Statistics were carried out by SPSS 20.0 software (SPSS Inc) or Graph Pad Prism 8.0.2 (GraphPad Software). The differences between two groups were calculated by Student's *t* test. One‐way ANOVA was used for the comparison of multiple groups. Mann‐Whitney test was performed in the analysis of circVRK1 expression between NP and PE placenta tissues, 24‐hour proteinuria quantification and proteinuria level. Birth gender was tested using the chi‐square test. *P* < 0.05 was represented as statistical significance.

## 3 RESULTS

### 3.1 Differential circRNA expression profiles in pre‐eclampsia and bioinformatical analysis

**FIGURE 1:**
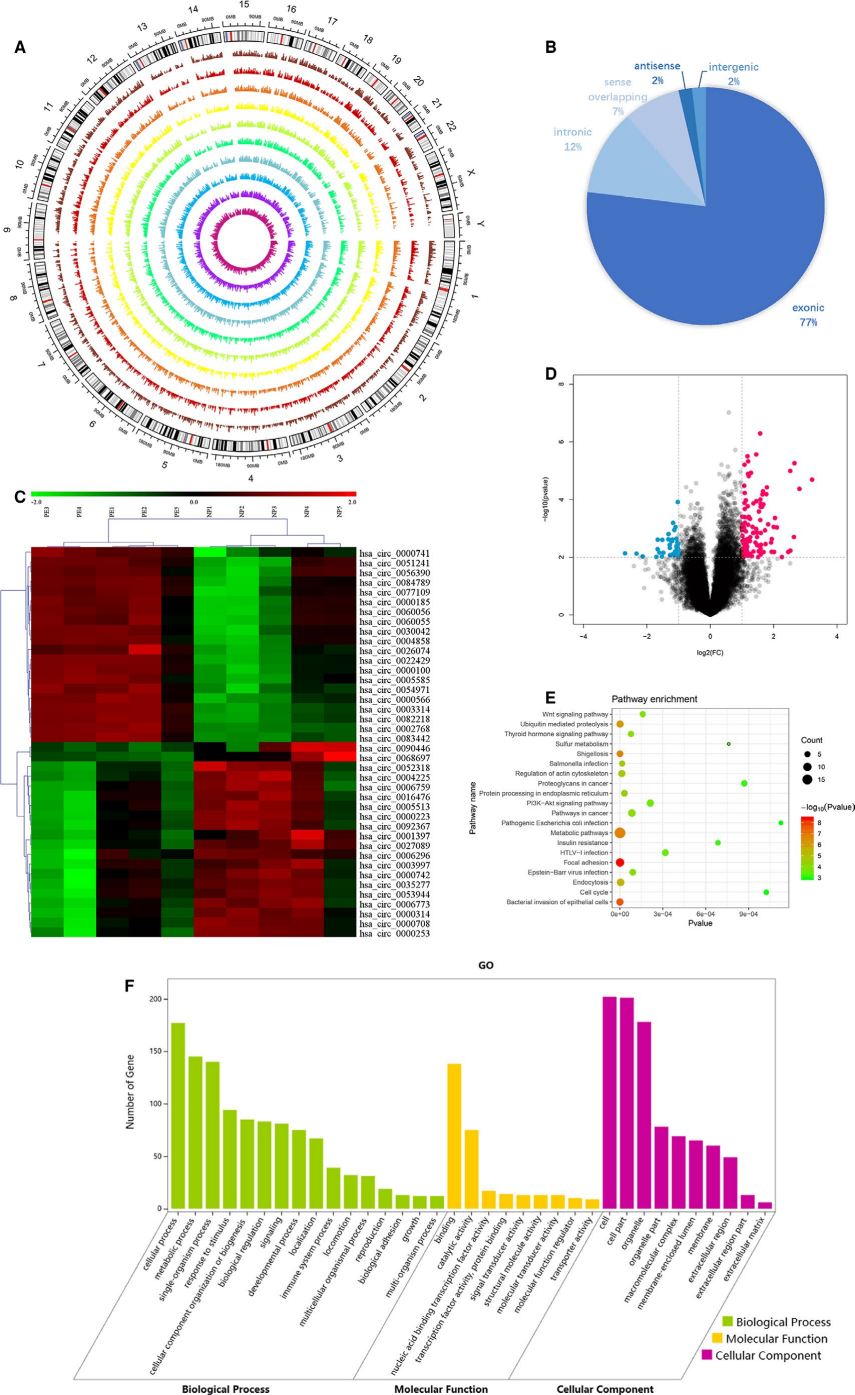
Analysis of microarray profile between PE and NP. A, Circos plot showing the differentially expressed circRNAs in human chromosomes (hg19). From the outside to inside represents NP 1‐5 and PE 1‐5. The values represent the log2 (fold change) of the two groups. B, Pie chart showing circRNA classes. C, Volcano plot of circRNAs. The red and blue points demonstrate the differentially expressed circRNAs with a greater than a two fold change. D, Cluster heat map of differentially expressed circRNAs with a greater than a twofold change in five paired PE and NP tissues. Red represents the up‐regulated circRNAs, and green represents the down‐regulated circRNAs. E, KEGG pathway enrichment of the parental genes of the differentially expressed circRNAs. The top 20 categories are exhibited. F, GO analysis of the parental genes of the differentially expressed circRNAs [Colour figure can be viewed at wileyonlinelibrary.com]

To explore the differentially expressed circRNAs in PE and NP tissues, microarray analysis was performed on pre‐eclampsia placental tissues (PE, N = 5) and normal pregnancy tissues (NP, N = 5). The circRNA microarray profile data have been loaded to the GEO database (GSE137854). The data left generated or used during the study appear in the submitted article. The locations on human chromosomes and the differential expression levels of these circRNAs are presented in Figure 1A. As the profile result showed, 77% of the differentially expressed circRNAs were exonic, 2% were intergenic, 7% were sense overlapping, 12% were intronic, and 2% were antisense (Figure 1B). The volcano plot showed the raw alterations in the circRNA expression pattern in the PE and NP placental tissues (Figure 1C). Hierarchical clustering analysis showed the top 20 up and down‐regulated circRNAs in the two groups (Figure 1D). Their information was shown in Table 2. In total, 273 differentially expressed circRNAs were screened from 13 617 circRNAs ranked by fold change ≥2.0. In the screened circRNAs, 182 were up‐regulated, and 91 were down‐regulated. 214 were annotated, and 58 were not matched with the circBase database.

**TABLE 2:** The top 20 up‐regulated and down‐regulated circRNAs in the profile

Target ID	Up/down	*P*‐value	Fold change	Chromosome	Spliced length (bp)	Type
hsa_circ_0003314	Up	2.03927E−05	9.2900555	chr1	333	Exonic
hsa_circ_0082218	Up	4.24988E−05	7.0404657	chr7	190	Exonic
hsa_circ_0002768	Up	5.51837E−06	6.3016641	chr3	376	Exonic
hsa_circ_0026074	Up	.001979575	6.2405526	chr12	3537	Exonic
hsa_circ_0084789	Up	.005926081	5.7689828	chr8	3139	Exonic
hsa_circ_0000185	Up	.016369772	5.7435394	chr1	235	Sense overlapping
hsa_circ_0077109	Up	.00991516	4.7965505	chr6	5178	Exonic
hsa_circ_0030042	Up	.011295009	4.3549539	chr13	1352	Exonic
hsa_circ_0022429	Up	.000924152	4.3000401	chr11	18 614	Exonic
hsa_circ_0000566	Up	.000434453	4.117601	chr14	1073	Exonic
hsa_circ_0000741	Up	.006584884	4.035136	chr17	336	Exonic
hsa_circ_0000100	Up	.000890969	3.9971006	chr1	393	Exonic
hsa_circ_0005585	Up	.003105024	3.7880943	chr5	623	Sense overlapping
hsa_circ_0060056	Up	.01587749	3.6704261	chr20	1018	Sense overlapping
hsa_circ_0051241	Up	.020328415	3.6653865	chr19	849	Exonic
hsa_circ_0060055	Up	.025061163	3.5921853	chr20	906	Sense overlapping
hsa_circ_0056390	Up	.043538103	3.572366	chr2	264	Exonic
hsa_circ_0004858	Up	.012141469	3.5709516	chr20	1521	Exonic
hsa_circ_0054971	Up	.000721003	3.5471022	chr2	273	Exonic
hsa_circ_0083442	Up	3.76527E−05	3.4562111	chr8	174	Exonic
hsa_circ_0006773	Down	.015148285	−2.872441	chr7	393	Exonic
hsa_circ_0003997	Down	.048612853	−2.9284819	chr11	493	Exonic
hsa_circ_0006296	Down	.026863443	−2.9351019	chr1	302	Exonic
hsa_circ_0035277	Down	.047902531	−2.9407083	chr15	223	Exonic
hsa_circ_0000742	Down	.023725357	−2.9421178	chr17	106	Intronic
hsa_circ_0090446	Down	.047031513	−3.0171368	chrX	916	Exonic
hsa_circ_0001397	Down	.006944568	−3.0399968	chr4	202	Exonic
hsa_circ_0068697	Down	.01389048	−3.085743	chr3	749	Exonic
hsa_circ_0005513	Down	.017308599	−3.0977857	chr7	357	Exonic
hsa_circ_0027089	Down	.002463823	−3.1496089	chr12	153	Exonic
hsa_circ_0052318	Down	.005249693	−3.1604508	chr19	2425	Exonic
hsa_circ_0006759	Down	.037097639	−3.1699524	chr2	78 995	Exonic
hsa_circ_0004225	Down	.00984201	−3.2501646	chr3	336	Exonic
hsa_circ_0053944	Down	.047609313	−3.4743432	chr2	1783	Exonic
hsa_circ_0016476	Down	.02598597	−3.8690257	chr1	7238	Exonic
hsa_circ_0000314	Down	.011769328	−4.0827121	chr11	126	Intergenic
hsa_circ_0000223	Down	.010104326	−4.615542	chr10	119	Intronic
hsa_circ_0000708	Down	.00748478	−5.0060877	chr16	129	Intronic
hsa_circ_0092367	Down	.020080124	−5.3031407	chr15	1180	Intronic
hsa_circ_0000253	Down	.00731473	−6.4449332	chr10	138	Intronic

Kyoto Encyclopedia of Genes and Genomes (KEGG) pathway and GO enrichment analysis was performed to investigate the potential roles of the parental genes related to the differentially expressed circRNAs (Figure 1E,F). KEGG pathway analysis revealed that the top three enriched pathways involved: metabolic pathways, focal adhesion and endocytosis. Moreover, these altered circRNAs may also impact several vital pathways that have a strong relationship with the PE progression, such as the phosphoinositide 3‐kinase (PI3K)/Akt signalling pathway^21^, ^22^, ^23^ and the Wnt signalling pathway.^24^, ^25^, ^26^ GO assessment involved three major domains: biological process (BP), molecular function (MF) and cellular component (CC). The top three enriched GO terms in each domain were cellular process, metabolic process and single‐organism process in BP; binding, catalytic activity and nucleic acid binding transcription factor activity in MF; cell, cell part, and organelle in CC.

### 3.2 Identification of circVRK1 in PE

**FIGURE 2:**
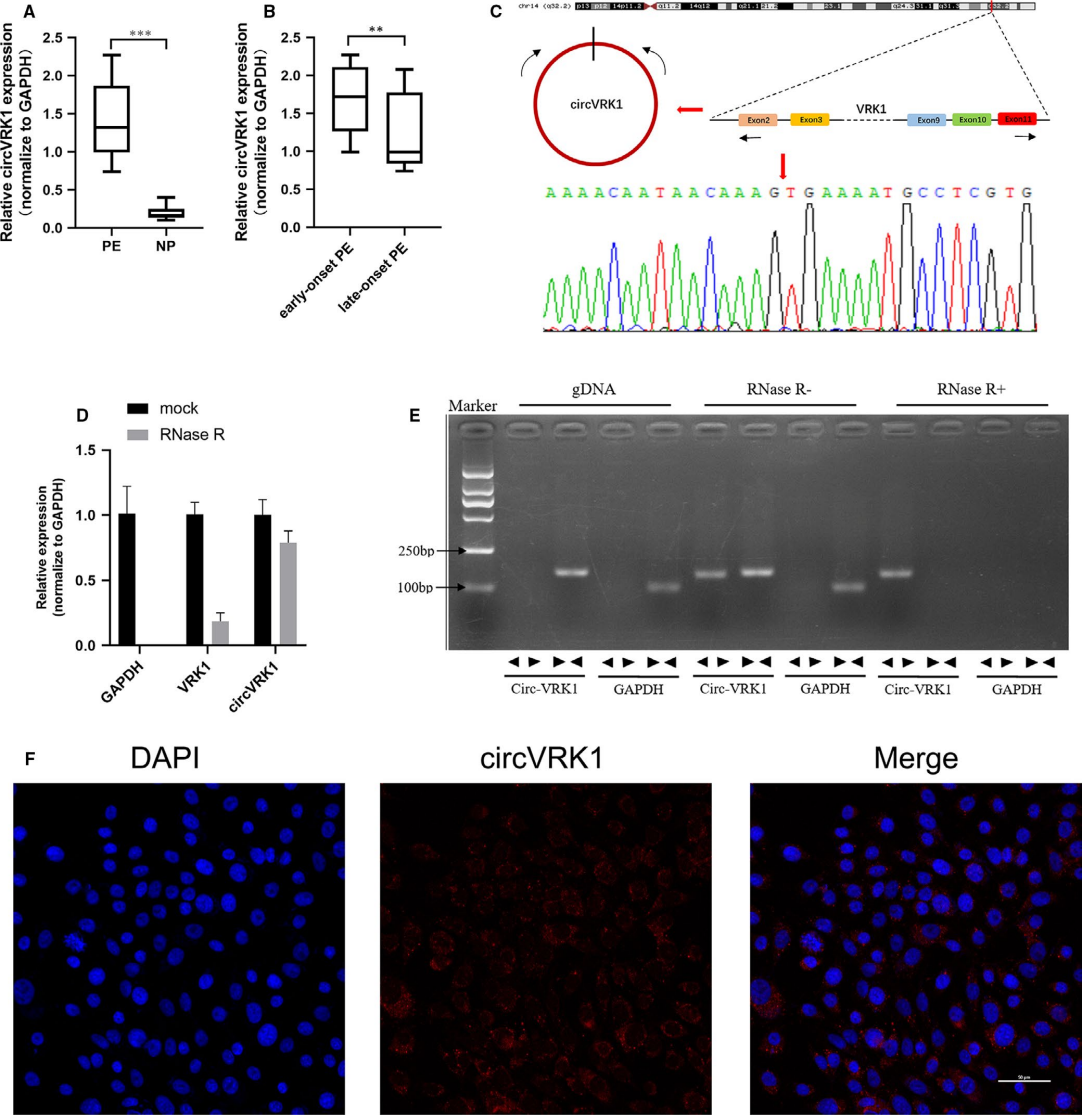
The identification of circVRK1. A, qPCR examined circVRK1 expression between PE and NP. B, qPCR examined circVRK1 expression between early‐onset PE, late‐onset PE. C, Sanger sequence examined the circular splicing site of circVRK1. D, qPCR examined circVRK1 expression. VRK1 was digested by RNase R but circVRK1 was not. E, Using convergent or divergent primers, circVRK was amplified in cDNA but not in gDNA by using divergent primers. F, Fluorescence in situ hybridization showed circVRK1 was widely expressed in the cytoplasm of HTR‐8/SVneo cells, the red represents circVRK1. Data are presented as means ± SD. **P < 0.01, ***P < 0.001 [Colour figure can be viewed at wileyonlinelibrary.com]

In the data of the circRNA microarray profile, according to the fold change and the *P*‐value, circVRK1 (hsa_circ_0000566) has drawn our attention. It is formed from exon 2 to 11 of the vaccinia‐related kinase 1 (VRK1) located on chromosome 14 (circbase: 97299803‐97327072). To verify the profile data, we measured circVRK1 expression in PE and NP placentas. (The primers applied in this experiment are shown in Table 3). The circVRK1 expression level was remarkably higher in the PE group than the NP (Figure 2A). Moreover, we also tested the expression between early‐onset PE and late‐onset PE. The results showed that the circVRK1 in the early‐onset PE group is remarkedly higher than the late‐onset PE group (Figure 2B). Early‐onset PE is believed to be a placental origin complication, which is caused by trophoblast cell dysfunction and insufficient spiral artery remodelling.^27^, ^28^ It was supposed that circVRK1 may be associated with trophoblast cell behaviours. Afterwards, we identified the specific back‐spliced circular construction of circVRK1. Firstly, we performed qPCR in HTR‐8/SVneo cells and Sanger sequencing for the products; the sequence result could completely match with the back‐spliced region (Figure 2C). Subsequently, we found that circVRK1 could resist the digestion of RNase R while VRK1 and GAPDH were digested by RNase R, and only amplified in cDNA by divergent primers but not in gDNA (Figure 2D,E). Fluorescence in situ hybridization analysis was performed to further explore the location of circVRK1, it was found expressed in distributed in the cytoplasm (Figure 2F). Taken together, the data suggested that the aberrantly expressed circVRK1 is correlated with the progression of PE and the trophoblast cell biological function.

**TABLE 3:** Primers for qPCR

Name	Forward (5′‐3′)	Reverse (5′‐3′)
GAPDH	GGGAAACTGTGGCGTGAT	GAGTGGGTGTCGCTGTTGA
U6	AACGCTTCACGAATTTGCGT	CTCGCTTCGGCAGCACA
circVRK1	ATTGGACCTCAGTGTTGTGGA	CAAATTGTTCTGCAAGATGTCTC
VRK1	TGCGAGGTGGAAGTAATGATTAAA	TCACAAACACACGGCTTTGG
miR‐221‐3p	GCGAAAGTGCTGCGACATTT	CGCGAGCTACATTGTCTGCTG
PTEN	TGGATTCGACTTACACTTGACCT	GGTGGGTTATGGTCTTCAAAAGG

### 3.3 Silencing circVRK1 promotes trophoblast cell migration, invasion and EMT

**FIGURE 3:**
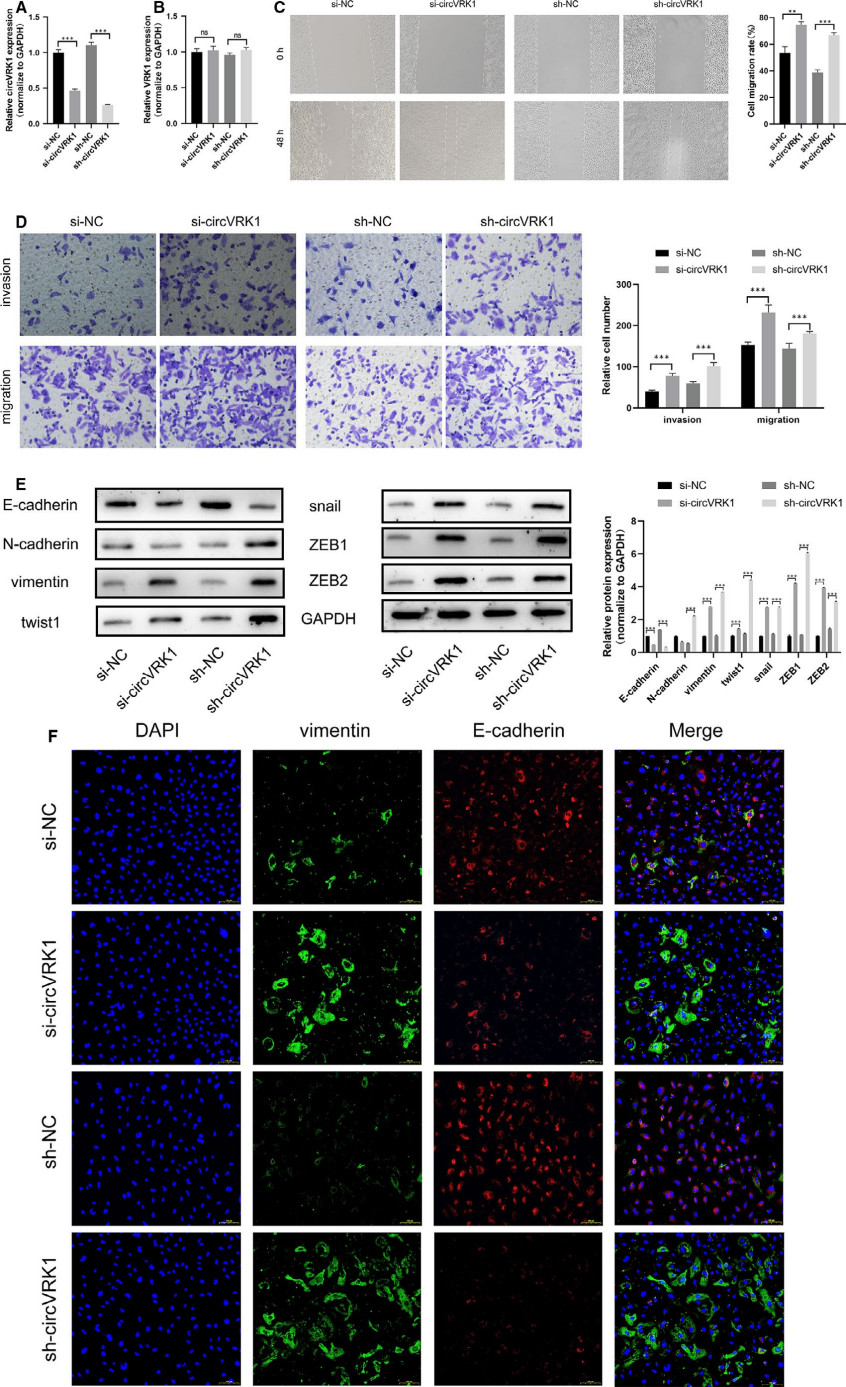
Silencing circVRK1 promoted trophoblast cell migration, invasion and EMT. A and B, qPCR was used to term that the siRNA and the shRNA were specific to circVRK1 but not VRK1. C, Wound assay was used to detect cell migration ability after cells were transfected with circVRK1 knockdown and NC. D, Transwell assays were performed to detect cell migration and invasion abilities in HTR‐8/Svneo cells. E, Western blot assay was used to evaluate the EMT‐related proteins in HTR‐8/Svneo cells. F, Immunofluorescence analysis was used to detect E‐cadherin and vimentin in HTR‐8/Svneo cells. Data are presented as means ± SD. **P < 0.01, ***P < 0.001, ns: no significance [Colour figure can be viewed at wileyonlinelibrary.com]

To investigate the potential roles of circVRK1. We successfully suppressed the circVRK1 expression in HTR‐8/SVneo cells using siRNA and shRNA (Figure S1A,B), which were specific to circVRK1 but not VRK1 (Figure 3A,B). From the wound assay, the cell migration rate in the circVRK1 knockdown group is higher than the NC group, which indicated circVRK1 knockdown promoted the migration of HTR‐8/SVneo cells (Figure 3C). Transwell assay illustrated that the silencing of circVRK1 significantly enhanced cell migration and invasion viabilities (Figure 3D). The EMT‐related proteins which were considered as EMT marker were detected by Western blot.^29^, ^30^, ^31^, ^32^, ^33^ As shown in Figure 3E, the vimentin, twist1, snail, ZEB1, ZEB2 expression in both circVRK1 knockdown group were remarkably elevated, while the E‐cadherin expression was down‐regulated. Furthermore, immunofluorescence staining confirmed that circVRK1 knockdown increased vimentin expression and decreased E‐cadherin expression, which further proved that circVRK1 knockdown promoted EMT of HTR‐8/SVneo cells (Figure 3F). Consequently, these data proved circVRK1 suppresses trophoblast cell migration, invasion and EMT.

### 3.4 circVRK1 functions as ceRNA to miR‐221‐3p trophoblast cell

**FIGURE 4:**
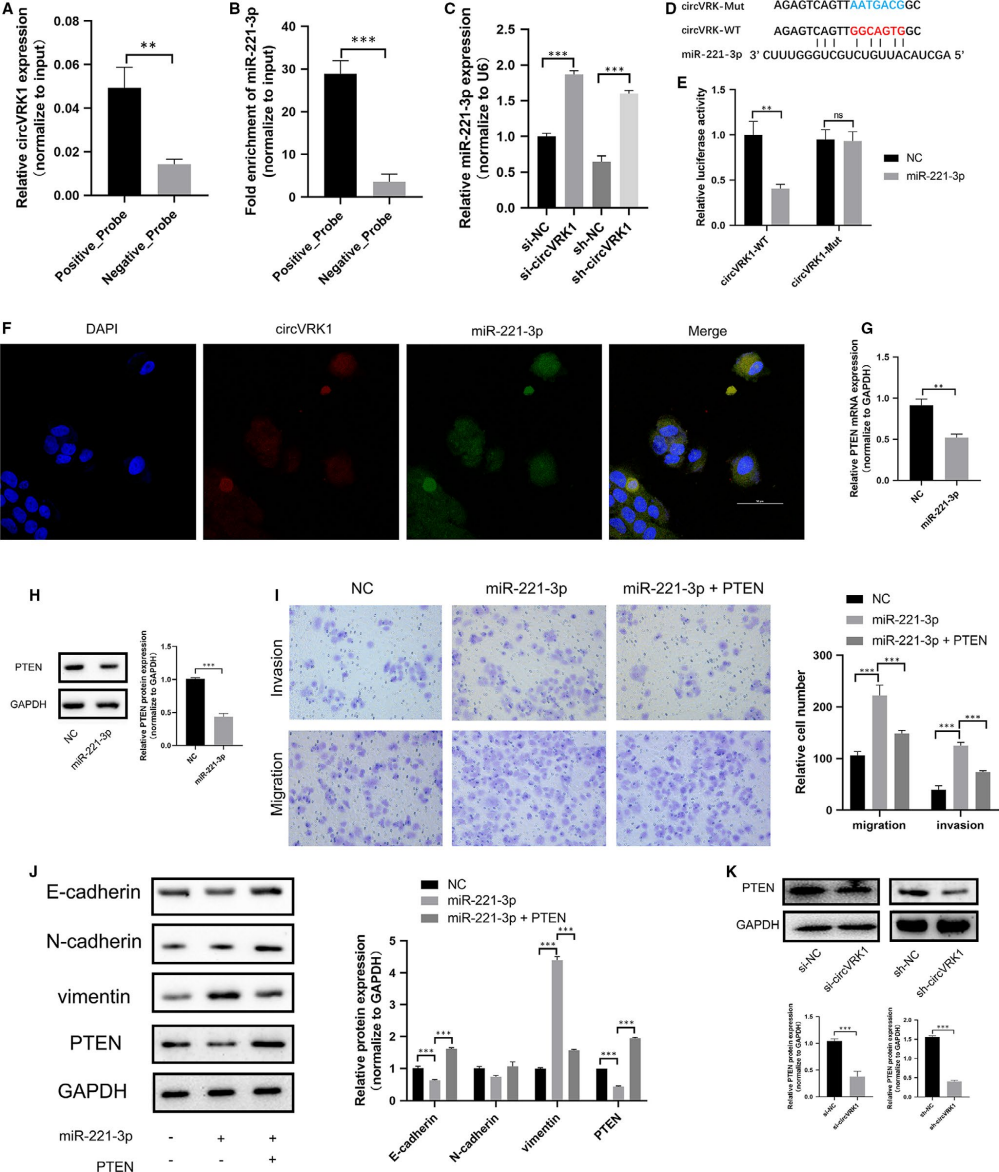
circVRK1 directly targeted to miR‐221‐3p and miR‐221‐3p promote trophoblast cell migration, invasion and EMT through PTEN. A and B, qPCR was used to determine the pull‐down efficiency and miR‐221‐3p with biotin‐labelled circVRK1 from HTR‐8/Svneo cells. C, qPCR was used to detect the miR‐221‐3p expression by circVRK1 knockdown. D, Schematic graph of wild‐type and mutated miR‐221‐3p binding sites of circVRK1. E, Luciferase assay was used to detect the wild‐type or mutated circVRK1 activity in miR‐221‐3p overexpression or NC. F, Fluorescence in situ hybridization showed circVRK1 was widely expressed in the cytoplasm of HTR‐8/SVneo cells, the red represented circVRK1, and the green represented miR‐221‐3p. G and H, qPCR and Western blot were used to determine the mRNA and protein expression by miR‐221‐3p overexpression. I, Transwell assays were performed to detect cell migration and invasion abilities. J, Western blot was used to evaluate E‐cadherin, N‐cadherin, vimentin and PTEN in HTR‐8/Svneo cells. K, Western blot was used to evaluate PTEN after circVRK1 knockdown in HTR‐8/Svneo cells. Data are presented as means ± SD. **P < 0.01, ***P < 0.001, ns: no significance [Colour figure can be viewed at wileyonlinelibrary.com]

According to the ceRNA theory, circRNA can sponge to miRNA to suppress the mRNA which shares the same binding sites, and the following translation.^14^ We further investigate the mechanism of the regulation of circVRK1 to trophoblast cells. RNA pull‐down and RNA sequencing were performed to detect the miRNA which has a potential binding relationship to circVRK1. The top 20 enriched miRNAs were represented in Table S2 ranked by fold change ≥2.0 and *P* < 0.001. Combining with the RNA pull‐down data and bioinformatic prediction, we selected a microRNA (hsa‐miR‐221‐3p, miR‐221‐3p). The result of RNA pull‐down showed that circVRK1 was successfully pulled down by miR‐221‐3p (Figure 4A,B). We further performed qPCR to find that the expression of miR‐221‐3p was remarkedly up‐regulated by circVRK1 knockdown (Figure 4C). RNA22 v2 and RNA hybrid were used to predict the binding relationship between circVRK1 and miR‐221‐3p (Figure 4D). Dual‐luciferase reporter assay showed that overexpressed miR‐221‐3p could significantly inhibit the luciferase activity of the wild‐type of circVRK1 but not the mutant (Figure 4E). Additionally, fluorescence in situ hybridization represented that circVRK1 and miR‐221‐3p were widely expressed in HTR‐8/SVneo cells (Figure 4F). Collectively, the results proved circVRK1 directly interacted with miR‐221‐3p in trophoblast cells.

### 3.5 miR‐221‐3p regulates the migration, invasion and EMT of trophoblast cell via regulating PTEN

By using TargetScan, we found phosphatase and tensin homolog (PTEN) could have the potential complementary sequences of miR‐221‐3p. Through qPCR and Western blot, we found that the mRNA and the protein expression of PTEN were down‐regulated by miR‐221‐3p overexpression (Figure 4G,H). As the functional experiments showed, overexpressed miR‐221‐3p remarkedly promoted HTR‐8/SVneo cell migration and invasion, and partly attenuated by PTEN (Figure 4I). Western blot assay revealed that miR‐221‐3p overexpression significantly up‐regulated vimentin, and down‐regulated E‐cadherin, while the N‐cadherin expression showed no significant difference between miR‐221‐3p and NC group. The E‐cadherin expression in the miR‐221‐3p + PTEN group was higher than the miR‐221‐3p group, and the result of vimentin showed the opposite, the PTEN expression in the cotransfection group was higher than the miR‐221‐3p mimics group (Figure 4J). Western blot showed that PTEN expression was down‐regulated by circVRK1 knockdown (Figure 4K). The results above demonstrated that miR‐221‐3p can promote trophoblast cell migration, invasion and EMT progression via regulating PTEN.

### 3.6 circVRK1 serves as ceRNA to inhibit trophoblast cell migration, invasion and EMT by sponging miR‐221‐3p to regulate PTEN

**FIGURE 5:**
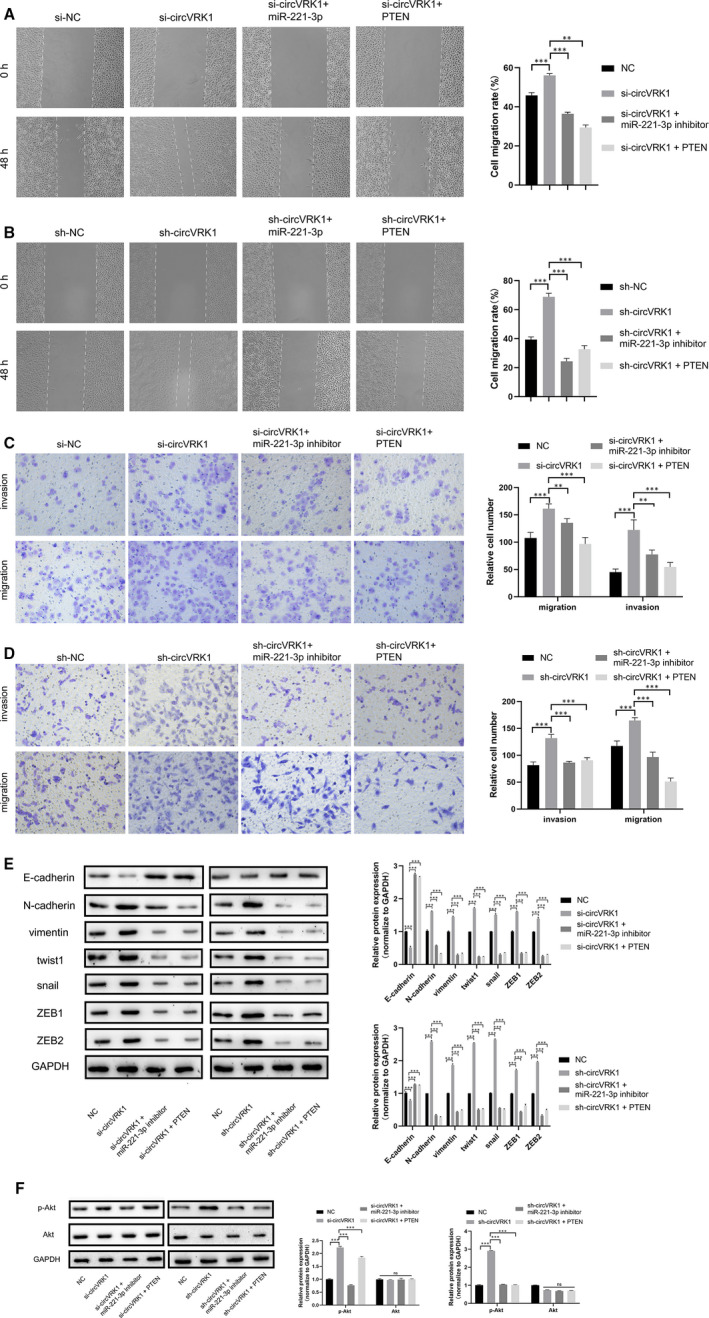
circVRK1 modulate trophoblast cell migration, invasion and EMT via regulating miR‐221‐3p/PTEN/Akt. A and B, Wound assay was performed cell migration ability of HTR‐8/Svneo cell. C and D, Transwell assays were used to detect cell migration and invasion abilities in HTR‐8/Svneo cells. E, Western blot assay was used to evaluate EMT‐related proteins in HTR‐8/Svneo cells. F, Western blot assay was used to evaluate p‐Akt and Akt in HTR‐8/Svneo cells. Data are presented as means ± SD. **P < 0.01, ***P < 0.001, ns: no significance [Colour figure can be viewed at wileyonlinelibrary.com]

To further explore whether circVRK1 suppresses trophoblast cell progression via miR‐221‐3p/PTEN in PE. Rescue experiments were performed by cotransfection of circVRK1 knockdown, miR‐221‐3p inhibitor and PTEN. As the results revealed, circVRK1 knockdown significantly promoted the migration and invasion of HTR‐8/SVneo cells, circVRK1 knockdown + miR‐221‐3p inhibitor greatly counteracted this phenomenon, besides, circVRK1 knockdown + PTEN also reversed this promotive effect (Figure 5A‐D). E‐cadherin was down‐regulated by circVRK1 knockdown, while N‐cadherin, vimentin, twist1, snail and ZEB1/2 were up‐regulated. The regulation of these proteins was counteracted by the cotransfection of miR‐221‐3p inhibitor or PTEN (Figure 5E). The results above proved circVRK1 sponges miR‐221‐3p to modulate trophoblast cell migration and invasion viabilities via regulating PTEN.

Moreover, PTEN is a well‐known tumour suppressor that has been proved closely related to the PI3K/Akt signal pathway.^34^, ^35^, ^36^, ^37^ So we further explore the potential regulation of circVRK1/miR‐221‐3p/PTEN axis in the PI3K/Akt activation. As the Western blot assay showed, the Akt expression showed no significant difference, circVRK1 knockdown showed higher phosphorylation levels of Akt. The cotransfection of miR‐221‐3p inhibitor or PTEN significantly weakened that effect (Figure 5F). These data indicated that the circVRK1/miR‐221‐3p/PTEN axis plays an important role in the PI3K/Akt signal pathway in trophoblast cell progression.

## 4 DISCUSSION

**FIGURE 6:**
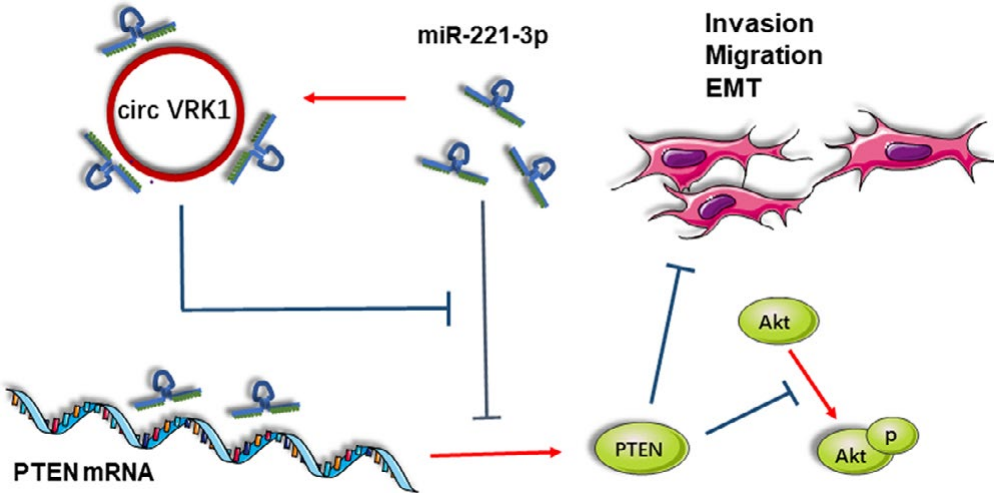
circVRK1 acts as a ceRNA to miR‐221‐3p to modulate trophoblast cell migration, invasion, and EMT via regulating PTEN [Colour figure can be viewed at wileyonlinelibrary.com]

Circular RNAs have gained considerable attention in cancer‐related or non‐cancer diseases in the past decades. Accumulating evidence has demonstrated that circRNAs are not simply byproducts of misspliced RNAs or splicing errors.^11^, ^38^ They are a class of covalently closed continuous loops RNAs that can tolerate RNase R digestion so that they are considered better diagnostic markers than linear transcripts.^39^ CircRNAs have been well characterized as hub genes, oncogenes or tumour suppressors in various diseases such as sciatic nerve injury,^15^ Alzheimer's disease,^40^ hepatocellular carcinoma,^16^, ^41^ and breast cancer.^42^ However, little information has been noted between circRNAs and PE. In the past 2 years, the researches of circular RNA in pre‐eclampsia ushered in a wave of climax, several circRNA were found out and were considered as potential clinical diagnostic markers or therapeutic targets.^18^, ^43^, ^44^, ^45^ In the study, we performed microarray to screen 273 circRNAs which have statistically significant differential expression between the placentas of PE and NP. GO enrichment and KEGG pathway analysis were performed on the parental genes corresponding to the circRNAs. Several important functions and pathways were screened which were well‐identified participating in PE progression, for example binding, cellular process, metabolic process, focal adhesion, regulation of the actin cytoskeleton, the PI3K/Akt signalling pathway, the Wnt signalling pathway and the MAPK signalling pathway. Overall, the profile data provide wide insights to explore circRNAs deep in PE. From the microarray, we identified a novel RNA (circVRK1) which is highly expressed in PE placental tissues especially early‐onset PE and plays a vital regulative role in trophoblast cell progression. It acts as a miR‐221‐3p sponge to inhibit trophoblast cell migration, invasion and EMT via regulating PTEN, the circVRK1/miR‐221 3P/PTEN axis is also revolved in PI3K/AKT pathway (Figure 6).

Non‐coding RNA‐miRNA crosstalk is one of the classic models in competing endogenous networks, including lncRNAs, circRNAs and pseudogenes, which contain miRNA binding sites so that they can sequester miRNAs.^46^ Argonaute (Ago), and its activate RNA‐induced silencing complex (RISC), is characterized as the central component of the crosstalk.^47^ The non‐coding RNAs bind to RISCs and release part of miRNA‐targeted mRNA, enabling the mRNAs to acquire the abilities of transcription and translation. This ceRNA crosstalk regulation may be symmetrical or asymmetrical,^48^ and also depends on their relative abundance, stoichiometry, the number of shared MREs and indirect interactions.^46^ Due to the high stability of circRNAs, circRNAs‐ceRNA crosstalk is believed to be more effective than linear RNAs. Here, we found that circVRK1 served as a ceRNA to miR‐221‐3p to regulate PTEN. It has been reported that miR‐221‐3p can promote tumour progression by suppressing PTEN progression in gastric carcinoma.^49^ miR‐221‐3p has been proved down‐regulated in the placentas of PE and promotes trophoblast cell growth, invasion and migration.^50^ PTEN is characterized as a tumour suppressor.^37^, ^51^, ^52^, ^53^ Its ectopic expression has been proved to be associated with trophoblastic diseases such as epithelioid trophoblastic tumour,^54^ hydatidiform mole,^55^, ^56^ early spontaneous abortion,^57^ and PE. It is expressed in cytotrophoblasts and syncytiotrophoblasts and is significantly elevated in the placentas of patients with PE.^58^, ^59^ In this study, we first proved that miR‐221‐3p can promote trophoblast cell EMT, as well as migration and invasion viabilities by inhibiting PTEN. The results of the pull‐down assay and the dual‐luciferase reporter assay proved the binding connection between circVRK1 and miR‐221‐3p. Combining the functional experiments, we finally proved that circVRK1 inhibits trophoblast cell migration, invasion and EMT by regulating miR‐221‐3p/PTEN. Furthermore, from the Western blot, circVRK1 knockdown also significantly elevated the phosphorylation of Akt, which indicated the inhibition of circVRK1 in PI3K/Akt activation. It is known that PI3K/Akt is widely involved in modulating cell biological behaviours such as migration and invasion abilities, as well as EMT.^60^, ^61^, ^62^ The PI3K/Akt signal pathway plays an important role in PE. PI3K/Akt activation, as well as the phosphorylation of Akt, remains at a low level during PE. The inhibition of Akt phosphorylation can lead to weakening migration and invasion viabilities.^21^, ^63^, ^64^ In the research, the phosphorylation of Akt was remarkedly regulated by circVRK1/miR‐221‐3p/PTEN axis, which termed that the regulation of the axis in trophoblast cell progression is involved in the PI3K/Akt signal pathway. However, there is little knowledge about that PI3K/Akt signal pathway modulating trophoblast cell EMT, and if the axis modulated the migration, invasion and EMT via the PI3K/Akt signal pathway in PE. It needs to deeper exploration in the future studies.

Back to circVRK1, it is spliced from VRK1 which is a Ser‐Thr kinase located in the nucleus which participates in the cell cycle containing DNA repair processes and apoptosis to DNA damage.^65^ It can specifically activate p53 phosphorylation to respond to DNA damage.^66^ Moreover, p53 remains at an elevated phosphorylation level in PE.^67^ It suggests that circVRK1 may also participate in the regulation of the trophoblast cell cycle. Next, we will further explore the influence on the trophoblast cell cycle from circVRK1 and VRK1.

In conclusion, this study demonstrates that circVRK1 can accelerate PE progression by suppressing trophoblast cell migration, invasion and EMT. It plays an inhibitory role by sponge miR‐221‐3p to regulate PTEN and the following PI3K/Akt signal pathway. Hoping this study can provide new therapeutic and diagnostic targets for PE.

## CONFLICT OF INTEREST

There is no conflict of interest.

## AUTHOR CONTRIBUTION

**Ziwei Li:** Conceptualization (lead); Data curation (lead); Formal analysis (lead); Investigation (equal); Methodology (lead); Project administration (lead); Validation (lead); Writing‐original draft (lead); Writing‐review & editing (equal). **Xinyi Zhou:** Formal analysis (supporting); Investigation (supporting); Methodology (supporting); Software (equal); Writing‐review & editing (equal). **Wenyan Gao:** Investigation (supporting). **Manni Sun:** Investigation (supporting). **Haiying Chen:** Investigation (supporting). **Tao Meng:** Funding acquisition (lead); Methodology (equal); Project administration (equal); Resources (lead).

## Supporting information

Supplementary Material

## Data Availability

The microarray profile data that support the findings of this study are openly available in the GEO database (GSE137854). The data not mentioned in the study that support the findings are available from the corresponding author upon reasonable request.

## References

[1] ACOG Practice Bulletin No. 202: gestational hypertension and preeclampsia. Obstet Gynecol. 2019;133(1):e1‐e25. 10.1097/AOG.0000000000003018 30575675

[2] Magee LA , Pels A , Helewa M , et al. The hypertensive disorders of pregnancy (29.3). Best Pract Res Clin Obstet Gynaecol. 2015;29(5):643‐657. 10.1016/j.bpobgyn.2015.04.001 26141795

[3] Huppertz B . Placental origins of preeclampsia: challenging the current hypothesis. Hypertension. 2008;51(4):970‐975. 10.1161/HYPERTENSIONAHA.107.107607 18259009

[4] Ji L , Brkic J , Liu M , Fu G , Peng C , Wang YL . Placental trophoblast cell differentiation: physiological regulation and pathological relevance to preeclampsia. Mol Aspects Med. 2013;34(5):981‐1023. 10.1016/j.mam.2012.12.008 23276825

[5] Lyall F , Robson SC , Bulmer JN . Spiral artery remodeling and trophoblast invasion in preeclampsia and fetal growth restriction: relationship to clinical outcome. Hypertension. 2013;62(6):1046‐1054. 10.1161/HYPERTENSIONAHA.113.01892 24060885

[6] Staff AC , Dechend R , Pijnenborg R . Learning from the placenta: acute atherosis and vascular remodeling in preeclampsia‐novel aspects for atherosclerosis and future cardiovascular health. Hypertension. 2010;56(6):1026‐1034. 10.1161/HYPERTENSIONAHA.110.157743 20956732

[7] Kalluri R , Weinberg RA . The basics of epithelial‐mesenchymal transition. J Clin Invest. 2009;119(6):1420‐1428. 10.1172/JCI39104 19487818 PMC2689101

[8] E. Davies J , Pollheimer J , Yong HEJ , et al. Epithelial‐mesenchymal transition during extravillous trophoblast differentiation. Cell Adh Migr. 2016;10(3):310‐321. 10.1080/19336918.2016.1170258 27070187 PMC4951171

[9] Nigro JM , Cho KR , Fearon ER , et al. Scrambled exons. Cell. 1991;64(3):607‐613. 1991322 10.1016/0092-8674(91)90244-s

[10] Jeck WR , Sharpless NE . Detecting and characterizing circular RNAs. Nat Biotechnol. 2014;32(5):453‐461. 10.1038/nbt.2890 24811520 PMC4121655

[11] Chen LL , Yang L . Regulation of circRNA biogenesis. RNA Biol. 2015;12(4):381‐388. 10.1080/15476286.2015.1020271 25746834 PMC4615371

[12] Yang W , Liu Y , Gao R , Xiu Z , Sun T . Knockdown of cZNF292 suppressed hypoxic human hepatoma SMMC7721 cell proliferation, vasculogenic mimicry, and radioresistance. Cell Signal. 2019;60:122‐135. 10.1016/j.cellsig.2019.04.011 31028816

[13] Visci G , Tolomeo D , Agostini A , Traversa D , Macchia G , Storlazzi CT . CircRNAs and fusion‐circRNAs in cancer: new players in an old game. Cell Signal. 2020;75:109747. 10.1016/j.cellsig.2020.109747 32860952

[14] Hansen TB , Jensen TI , Clausen BH , et al. Natural RNA circles function as efficient microRNA sponges. Nature. 2013;495(7441):384‐388. 10.1038/nature11993 23446346

[15] Zhou ZB , Niu YL , Huang GX , Lu JJ , Chen A , Zhu L . Silencing of circRNA.2837 plays a protective role in sciatic nerve injury by sponging the miR‐34 family via regulating neuronal autophagy. Mol Ther Nucleic Acids. 2018;12:718‐729. 10.1016/j.omtn.2018.07.011 30098504 PMC6088565

[16] Gao S , Hu W , Huang X , et al. Circ_0001178 regulates miR‐382/VEGFA axis to facilitate hepatocellular carcinoma progression. Cell Signal. 2020;72:109621. 10.1016/j.cellsig.2020.109621 32240747

[17] Memczak S , Jens M , Elefsinioti A , et al. Circular RNAs are a large class of animal RNAs with regulatory potency. Nature. 2013;495(7441):333‐338. 10.1038/nature11928 23446348

[18] Shen XY , Zheng LL , Huang J , et al. CircTRNC18 inhibits trophoblast cell migration and epithelial‐mesenchymal transition by regulating miR‐762/Grhl2 pathway in pre‐eclampsia. RNA Biol. 2019;16(11):1565‐1573. 10.1080/15476286.2019.1644591 31354028 PMC6779405

[19] Zhou W , Wang H , Yang J , et al. Down‐regulated circPAPPA suppresses the proliferation and invasion of trophoblast cells via the miR‐384/STAT3 pathway. Biosci Rep. 2019;39(9). 10.1042/BSR20191965 PMC6732364 31427481

[20] von Dadelszen P , Magee LA , Roberts JM . Subclassification of preeclampsia. Hypertens Pregnancy. 2003;22(2):143‐148. 10.1081/PRG-120021060 12908998

[21] Cudmore MJ , Ahmad S , Sissaoui S , et al. Loss of Akt activity increases circulating soluble endoglin release in preeclampsia: identification of inter‐dependency between Akt‐1 and heme oxygenase‐1. Eur Heart J. 2012;33(9):1150‐1158. 10.1093/eurheartj/ehr065 21411816 PMC3341632

[22] Xu Y , Sui L , Qiu B , Yin X , Liu J , Zhang X . ANXA4 promotes trophoblast invasion via the PI3K/Akt/eNOS pathway in preeclampsia. Am J Physiol Cell Physiol. 2019;316(4):C481‐C491. 10.1152/ajpcell.00404.2018 30673304

[23] Xu J , Xia Y , Zhang H , Guo H , Feng K , Zhang C . Overexpression of long non‐coding RNA H19 promotes invasion and autophagy via the PI3K/AKT/mTOR pathways in trophoblast cells. Biomed Pharmacother. 2018;101:691‐697. 10.1016/j.biopha.2018.02.134 29522949

[24] Schlosser K , Kaur A , Dayan N , Stewart DJ , Pilote L , Delles C . Circulating miR‐206 and Wnt‐signaling are associated with cardiovascular complications and a history of preeclampsia in women. Clin Sci. 2020;134(2):87‐101. 10.1042/CS20190920 PMC8299351 31899480

[25] Li L , Peng W , Zhou Q , Wan JP , Wang XT , Qi HB . LRP6 regulates Rab7‐mediated autophagy through the Wnt/beta‐catenin pathway to modulate trophoblast cell migration and invasion. J Cell Biochem. 2020;121(2):1599‐1609. 10.1002/jcb.29394 31544984

[26] Knofler M , Pollheimer J . Human placental trophoblast invasion and differentiation: a particular focus on Wnt signaling. Front Genet. 2013;4:190. 10.3389/fgene.2013.00190 24133501 PMC3783976

[27] Herzog EM , Eggink AJ , Reijnierse A , et al. Impact of early‐ and late‐onset preeclampsia on features of placental and newborn vascular health. Placenta. 2017;49:72‐79. 10.1016/j.placenta.2016.11.014 28012458

[28] Tayyar AT , Karakus R , Eraslan Sahin M , et al. Wnt signaling pathway in early‐ and late‐onset preeclampsia: evaluation with Dickkopf‐1 and R‐Spondin‐3 glycoproteins. Arch Gynecol Obstet. 2019;299(6):1551‐1556. 10.1007/s00404-019-05126-8 30905002

[29] DaSilva‐Arnold SC , Kuo CY , Davra V , et al. ZEB2, a master regulator of the epithelial‐mesenchymal transition, mediates trophoblast differentiation. Mol Hum Reprod. 2019;25(2):61‐75. 10.1093/molehr/gay053 30462321 PMC6497037

[30] Ma L , Zhang Z , Dong K , Ma Y . TWIST1 Alleviates hypoxia‐induced damage of trophoblast cells by inhibiting mitochondrial apoptosis pathway. Exp Cell Res. 2019;385(2):111687. 10.1016/j.yexcr.2019.111687 31669261

[31] Wu D , Shi L , Chen X , Cen H , Mao D . beta‐TrCP suppresses the migration and invasion of trophoblast cells in preeclampsia by down‐regulating Snail. Exp Cell Res. 2020;395(2):112230. 10.1016/j.yexcr.2020.112230 32781057

[32] Wu GM , Jin Y , Cao YM , Li JY . The diagnostic value and regulatory mechanism of miR‐200a targeting ZEB1 in pregnancy‐induced hypertension. Hypertens Pregnancy. 2020;39(3):243‐251. 10.1080/10641955.2020.1757700 32345067

[33] Yu L , Kuang LY , He F , et al. The role and molecular mechanism of long nocoding RNA‐MEG3 in the pathogenesis of preeclampsia. Reprod Sci. 2018;25(12):1619‐1628. 10.1177/1933719117749753 29361889

[34] Lee M‐S , Jeong M‐H , Lee H‐W , et al. PI3K/AKT activation induces PTEN ubiquitination and destabilization accelerating tumourigenesis. Nat Commun. 2015;6:7769. 10.1038/ncomms8769 26183061 PMC4518267

[35] Nakahata S , Ichikawa T , Maneesaay P , et al. Loss of NDRG2 expression activates PI3K‐AKT signalling via PTEN phosphorylation in ATLL and other cancers. Nat Commun. 2014;5:3393. 10.1038/ncomms4393 24569712 PMC3948061

[36] Li Y , Tsang CK , Wang S , et al. MAF1 suppresses AKT‐mTOR signaling and liver cancer through activation of PTEN transcription. Hepatology. 2016;63(6):1928‐1942. 10.1002/hep.28507 26910647 PMC5021206

[37] Lee YR , Chen M , Pandolfi PP . The functions and regulation of the PTEN tumour suppressor: new modes and prospects. Nat Rev Mol Cell Biol. 2018;19(9):547‐562. 10.1038/s41580-018-0015-0 29858604

[38] Cocquerelle C , Mascrez B , Hetuin D , Bailleul B . Mis‐splicing yields circular RNA molecules. FASEB J. 1993;7(1):155‐160. 7678559 10.1096/fasebj.7.1.7678559

[39] Zhu Q , Lu G , Luo Z , et al. CircRNA circ_0067934 promotes tumor growth and metastasis in hepatocellular carcinoma through regulation of miR‐1324/FZD5/Wnt/beta‐catenin axis. Biochem Biophys Res Commun. 2018;497(2):626‐632. 10.1016/j.bbrc.2018.02.119 29458020

[40] Zhao Y , Alexandrov PN , Jaber V , Lukiw WJ . Deficiency in the ubiquitin conjugating enzyme UBE2A in Alzheimer's disease (AD) is linked to deficits in a natural circular miRNA‐7 sponge (circRNA; ciRS‐7). Genes. 2016;7(12):116. 10.3390/genes7120116 PMC5192492 27929395

[41] Zhen NI , Gu S , Ma JI , et al. CircHMGCS1 promotes hepatoblastoma cell proliferation by regulating the IGF signaling pathway and glutaminolysis. Theranostics. 2019;9(3):900‐919. 10.7150/thno.29515 30809316 PMC6376477

[42] Yang R , Xing L , Zheng X , Sun Y , Wang X , Chen J . The circRNA circAGFG1 acts as a sponge of miR‐195‐5p to promote triple‐negative breast cancer progression through regulating CCNE1 expression. Mol Cancer. 2019;18(1):4. 10.1186/s12943-018-0933-7 30621700 PMC6325825

[43] Zhang Y , Cao L , Jia J , et al. CircHIPK3 is decreased in preeclampsia and affects migration, invasion, proliferation, and tube formation of human trophoblast cells. Placenta. 2019;85:1‐8. 10.1016/j.placenta.2019.07.010 31421528

[44] Zhang Y , Yang H , Zhang Y , Shi J , Chen R , Xiao X . CircSFXN1 regulates the behaviour of trophoblasts and likely mediates preeclampsia. Placenta. 2020;101:115‐123. 10.1016/j.placenta.2020.09.012 32950919

[45] Zhang Y , Yang H , Zhang Y , Shi J , Chen R . circCRAMP1L is a novel biomarker of preeclampsia risk and may play a role in preeclampsia pathogenesis via regulation of the MSP/RON axis in trophoblasts. BMC Pregnancy Childbirth. 2020;20(1):652. 10.1186/s12884-020-03345-5 33109096 PMC7590488

[46] Tay Y , Rinn J , Pandolfi PP . The multilayered complexity of ceRNA crosstalk and competition. Nature. 2014;505(7483):344‐352. 10.1038/nature12986 24429633 PMC4113481

[47] Loinger A , Shemla Y , Simon I , Margalit H , Biham O . Competition between small RNAs: a quantitative view. Biophys J. 2012;102(8):1712‐1721. 10.1016/j.bpj.2012.01.058 22768926 PMC3328701

[48] Ala U , Karreth FA , Bosia C , et al. Integrated transcriptional and competitive endogenous RNA networks are cross‐regulated in permissive molecular environments. Proc Natl Acad Sci USA. 2013;110(18):7154‐7159. 10.1073/pnas.1222509110 23536298 PMC3645534

[49] Shi J , Zhang Y , Jin N , Li Y , Wu S , Xu L . MicroRNA‐221‐3p plays an oncogenic role in gastric carcinoma by inhibiting PTEN expression. Oncol Res. 2017;25(4):523‐536. 10.3727/096504016X14756282819385 27712596 PMC7841127

[50] Yang Y , Li H , Ma Y , Zhu X , Zhang S , Li J . MiR‐221‐3p is down‐regulated in preeclampsia and affects trophoblast growth, invasion and migration partly via targeting thrombospondin 2. Biomed Pharmacother. 2019;109:127‐134. 10.1016/j.biopha.2018.10.009 30396069

[51] Lee MF , Trotman LC . PTEN: bridging endocytosis and signaling. Cold Spring Harb Perspect Med. 2019;10(10). 10.1101/cshperspect.a036103 PMC7528861 31818848

[52] Kohnoh T , Hashimoto N , Ando A , et al. Hypoxia‐induced modulation of PTEN activity and EMT phenotypes in lung cancers. Cancer Cell Int. 2016;16:33. 10.1186/s12935-016-0308-3 27095949 PMC4836157

[53] Dai X , Guo X , Liu J , et al. Circular RNA circGRAMD1B inhibits gastric cancer progression by sponging miR‐130a‐3p and regulating PTEN and p21 expression. Aging. 2019;11(21):9689‐9708. 10.18632/aging.102414 31719211 PMC6874462

[54] Cho EJ , Chun SM , Park H , Sung CO , Kim KR . Whole transcriptome analysis of gestational trophoblastic neoplasms reveals altered PI3K signaling pathway in epithelioid trophoblastic tumor. Gynecol Oncol. 2020;157(1):151‐160. 10.1016/j.ygyno.2019.09.022 31954539

[55] Chen H , Ye D , Xie X , Lu W , Zhu C , Chen X . PTEN promoter methylation and protein expression in normal early placentas and hydatidiform moles. J Soc Gynecol Investig. 2005;12(3):214‐217. 10.1016/j.jsgi.2005.01.009 15784509

[56] Wang YX , Zhao JR , Xu YY , Wu WB , Zhang HJ . miR‐21 is overexpressed in hydatidiform mole tissues and promotes proliferation, migration, and invasion in choriocarcinoma cells. Int J Gynecol Cancer. 2017;27(2):364‐374. 10.1097/IGC.0000000000000861 27922982

[57] Tokyol C , Aktepe F , Husniye Dilek F , Yilmazer M . Comparison of placental PTEN and beta1 integrin expression in early spontaneous abortion, early and late normal pregnancy. Ups J Med Sci. 2008;113(2):235‐242. 10.3109/2000-1967-231 18509818

[58] Xiao J , Tao T , Yin Y , Zhao L , Yang L , Hu L . miR‐144 may regulate the proliferation, migration and invasion of trophoblastic cells through targeting PTEN in preeclampsia. Biomed Pharmacother. 2017;94:341‐353. 10.1016/j.biopha.2017.07.130 28772212

[59] Xue P , Fan W , Diao Z , et al. Up‐regulation of PTEN via LPS/AP‐1/NF‐kappaB pathway inhibits trophoblast invasion contributing to preeclampsia. Mol Immunol. 2020;118:182‐190. 10.1016/j.molimm.2019.12.018 31896494

[60] Sun F , Wang J , Sun Q , et al. Interleukin‐8 promotes integrin beta3 upregulation and cell invasion through PI3K/Akt pathway in hepatocellular carcinoma. J Exp Clin Cancer Res. 2019;38(1):449. 10.1186/s13046-019-1455-x 31684995 PMC6829822

[61] Xu J , Xiao Y , Liu B , et al. Exosomal MALAT1 sponges miR‐26a/26b to promote the invasion and metastasis of colorectal cancer via FUT4 enhanced fucosylation and PI3K/Akt pathway. J Exp Clin Cancer Res. 2020;39(1):54. 10.1186/s13046-020-01562-6 32209115 PMC7092616

[62] Karimi Roshan M , Soltani A , Soleimani A , Rezaie Kahkhaie K , Afshari AR , Soukhtanloo M . Role of AKT and mTOR signaling pathways in the induction of epithelial‐mesenchymal transition (EMT) process. Biochimie. 2019;165:229‐234. 10.1016/j.biochi.2019.08.003 31401189

[63] Chen J , Yue C , Xu J , et al. Downregulation of receptor tyrosine kinase‐like orphan receptor 1 in preeclampsia placenta inhibits human trophoblast cell proliferation, migration, and invasion by PI3K/AKT/mTOR pathway accommodation. Placenta. 2019;82:17‐24. 10.1016/j.placenta.2019.05.002 31174622

[64] Wang L , Zhang Y , Qu H , et al. Reduced ELABELA expression attenuates trophoblast invasion through the PI3K/AKT/mTOR pathway in early onset preeclampsia. Placenta. 2019;87:38‐45. 10.1016/j.placenta.2019.08.077 31546152

[65] Campillo‐Marcos I , Lazo PA . Implication of the VRK1 chromatin kinase in the signaling responses to DNA damage: a therapeutic target? Cell Mol Life Sci. 2018;75(13):2375‐2388. 10.1007/s00018-018-2811-2 29679095 PMC5986855

[66] Lopez‐Sanchez I , Valbuena A , Vazquez‐Cedeira M , et al. VRK1 interacts with p53 forming a basal complex that is activated by UV‐induced DNA damage. FEBS Lett. 2014;588(5):692‐700. 10.1016/j.febslet.2014.01.040 24492002

[67] Sharp AN , Heazell AE , Baczyk D , et al. Preeclampsia is associated with alterations in the p53‐pathway in villous trophoblast. PLoS One. 2014;9(1):e87621. 10.1371/journal.pone.0087621 24498154 PMC3907567

