# Supplementary material for: Circular RNA VRK1 facilitates pre‐eclampsia progression via sponging miR‐221‐3P to regulate PTEN/Akt
Source: J Cell Mol Med. 2021 Mar 18;26(6):1826–41. doi: 10.1111/jcmm.16454 (PMC8918405; doi:10.1111/jcmm.16454)

| **Supporting Table. 1 The antibodies in the study.** | | | |
| --- | --- | --- | --- |
| Name | Dilution ratio | Source | Identifier |
| E-cadherin | 1: 1000 (WB) | Cell Signaling Technology, USA | Cat# 14472S |
|  | 1: 50 (IHC) |  |  |
| N-cadherin | 1: 1500 (WB) | Cell Signaling Technology, USA | Cat# 13116S |
| vimentin | 1: 1000 (WB) | Cell Signaling Technology, USA | Cat# 5741S |
|  | 1: 100 (IHC) |  |  |
| ZEB1 | 1: 1000 (WB) | Cell Signaling Technology, USA | Cat# 70512S |
| ZEB2 | 1: 1000 (WB) | Cell Signaling Technology, USA | Cat# 97885S |
| snail | 1: 1000 (WB) | Cell Signaling Technology, USA | Cat# 3879S |
| twist1 | 1: 1000 (WB) | Cell Signaling Technology, USA | Cat# 69366S |
| Akt | 1: 1000 (WB) | Cell Signaling Technology, USA | Cat# 4961S |
| p-AKT | 1: 2000 (WB) | Cell Signaling Technology, USA | Cat# 4060S |
| GAPDH | 1: 1000 (WB) | Cell Signaling Technology, USA | Cat# 5174S |

WB: Western Blot, IHC: Immunofluorescence

| **Supporting Table. 2 The top 50 enriched miRNAs by RNA pull-down assay** | | | |
| --- | --- | --- | --- |
| **miRNA** | **Sequence** | **Fold change (positive / Input)** | **p-value**  **(positive / Input)** |
| hsa-miR-4443 | TTGGAGGCGTGGGTTTT | 12.80963079 | 9.95E-12 |
| hsa-miR-4488 | AGGGGGCGGGCTCCGGCG | 12.19964837 | 3.41E-36 |
| hsa-miR-1285-3p | TCTGGGCAACAAAGTGAGACCT | 10.36970111 | 5.79E-06 |
| hsa-miR-27b-5p | AGAGCTTAGCTGATTGGTGAAC | 8.006019242 | 2.11E-18 |
| hsa-miR-193b-5p | CGGGGTTTTGAGGGCGAGATGA | 6.709806603 | 1.13E-08 |
| hsa-miR-3195 | CGCGCCGGGCCCGGGTT | 6.557310998 | 9.22E-11 |
| hsa-miR-1180-3p | TTTCCGGCTCGCGTGGGTGTGT | 6.50647913 | 2.53E-08 |
| hsa-miR-1307-5p | TCGACCGGACCTCGACCGGCT | 6.099824184 | 0.002424935 |
| hsa-miR-615-3p | TCCGAGCCTGGGTCTCCCTCTT | 6.057756431 | 3.23E-21 |
| hsa-miR-193a-5p | TGGGTCTTTGCGGGCGAGATGA | 5.855831217 | 4.01E-11 |
| hsa-miR-320a-3p | AAAAGCTGGGTTGAGAGGGCGA | 5.520857821 | 1.31E-103 |
| hsa-let-7d-5p | AGAGGTAGTAGGTTGCATAGTT | 5.489841766 | 3.04E-42 |
| hsa-miR-132-3p | TAACAGTCTACAGCCATGGTCG | 5.489841766 | 5.15E-06 |
| hsa-miR-22-3p | AAGCTGCCAGTTGAAGAACTGT | 5.223344593 | 4.25E-163 |
| hsa-miR-181d-5p | AACATTCATTGTTGTCGGTGGGT | 4.574868138 | 1.45E-05 |
| hsa-miR-7704 | CGGGGTCGGCGGCGACGTG | 4.431418883 | 2.81E-184 |
| hsa-let-7c-5p | TGAGGTAGTAGGTTGTATGGTT | 4.360421194 | 7.46E-50 |
| hsa-miR-197-3p | TTCACCACCTTCTCCACCCAGC | 4.354012435 | 1.73E-12 |
| hsa-miR-3615 | TCTCTCGGCTCCTCGCGGCTC | 4.346124731 | 0.000218883 |
| hsa-let-7d-3p | CTATACGACCTGCTGCCTTTCT | 4.313447102 | 1.19E-06 |
| hsa-miR-196a-5p | TAGGTAGTTTCATGTTGTTGGG | 4.052026065 | 4.83E-06 |
| hsa-miR-125a-5p | TCCCTGAGACCCTTTAACCTGTGA | 3.939580772 | 1.26E-142 |
| hsa-miR-423-3p | AGCTCGGTCTGAGGCCCCTCAGT | 3.914978067 | 4.76E-102 |
| hsa-miR-99a-5p | AACCCGTAGATCCGATCTTGTG | 3.747141385 | 8.92E-229 |
| hsa-miR-125b-1-3p | ACGGGTTAGGCTCTTGGGAGCT | 3.72299614 | 1.52E-09 |
| hsa-miR-744-5p | TGCGGGGCTAGGGCTAACAGCA | 3.632168037 | 6.58E-19 |
| hsa-miR-181b-5p | AACATTCATTGCTGTCGGTGGGT | 3.461807437 | 1.98E-47 |
| hsa-miR-629-5p | TGGGTTTACGTTGGGAGAACT | 3.283877951 | 5.41E-17 |
| hsa-miR-4664-3p | CTTCCGGTCTGTGAGCCCCGTC | 3.137052438 | 0.013198907 |
| hsa-miR-589-5p | TGAGAACCACGTCTGCTCTGAG | 3.102954042 | 1.00E-05 |
| hsa-miR-22-5p | AGTTCTTCAGTGGCAAGCTTTA | 3.102954042 | 1.00E-05 |
| hsa-miR-98-5p | TGAGGTAGTAAGTTGTATTGTT | 3.061642523 | 2.69E-20 |
| hsa-miR-181a-5p | AACATTCAACGCTGTCGGTGAGT | 3.055586347 | 5.31E-40 |
| hsa-miR-155-5p | TTAATGCTAATCGTGATAGGGGTT | 2.987668988 | 7.27E-10 |
| hsa-miR-1303 | TTTAGAGACGGGGTCTTGCTCT | 2.956068643 | 0.001665625 |
| hsa-miR-221-3p | AGCTACATTGTCTGCTGGGTTTC | 2.904164081 | 3.58E-55 |
| hsa-miR-30a-3p | CTTTCAGTCGGATGTTTGCAGC | 2.836785705 | 8.23E-39 |
| hsa-miR-149-5p | TCTGGCTCCGTGTCTTCACTCCC | 2.836418246 | 0.000211388 |
| hsa-miR-16-2-3p | CCAATATTACTGTGCTGCTTTA | 2.805919125 | 7.74E-06 |
| hsa-miR-222-3p | AGCTACATCTGGCTACTGGGT | 2.744920883 | 2.61E-05 |
| hsa-miR-425-5p | AATGACACGATCACTCCCGTTGA | 2.714421762 | 1.37E-09 |
| hsa-miR-423-5p | TGAGGGGCAGAGAGCGAGACTTT | 2.68048612 | 4.97E-20 |
| hsa-let-7b-3p | CTATACAACCTACTGCCTTCCC | 2.661741462 | 0.000332929 |
| hsa-miR-502-3p | AATGCACCTGGGCAAGGATTCA | 2.643257147 | 0.022045971 |
| hsa-miR-146b-5p | TGAGAACTGAATTCCATAGGCTG | 2.598525103 | 1.70E-07 |
| hsa-miR-1307-3p | ACTCGGCGTGGCGTCGGTCGTG | 2.511168496 | 1.47E-16 |
| hsa-miR-193b-3p | AACTGGCCCTCAAAGTCCCGCT | 2.505284933 | 1.12E-10 |
| hsa-miR-345-5p | GCTGACTCCTAGTCCAGGGCTC | 2.401805773 | 0.000166045 |
| hsa-miR-95-3p | TTCAACGGGTATTTATTGAGCA | 2.378931432 | 3.54E-06 |
| hsa-miR-1246 | AATGGATTTTTGGAGCAGG | 2.378931432 | 0.003224225 |

Supporting Fig. (A and B) qPCR was used to term that si-2 and sh-3 have the highest transfection efficiency. (C) Western blot assay was used to evaluate EMT-related proteins after PTEN overexpression in HTR-8/Svneo cells. Data are presented as means ± SD. ** p < 0.01, *** p < 0.001
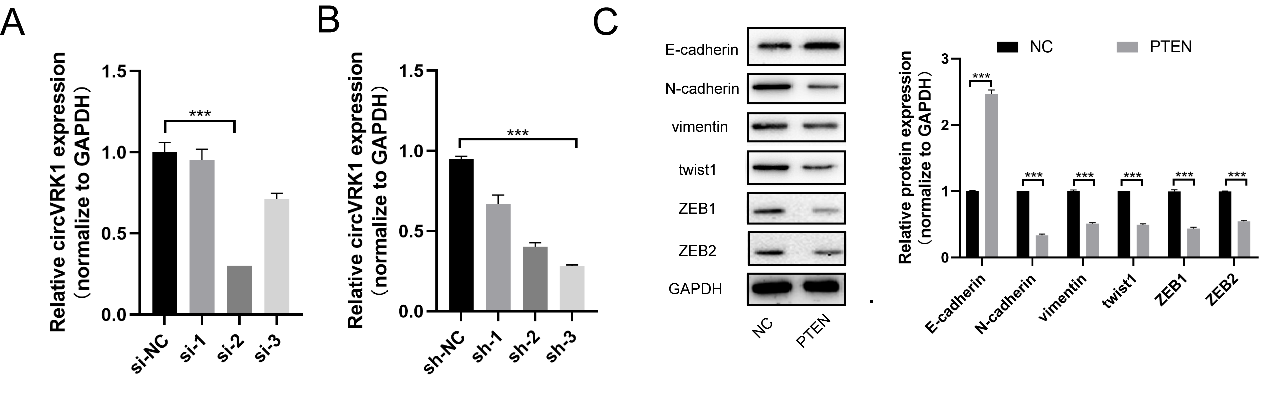

Supplement: Supplementary file 1 — Supplementary Material [file JCMM-26-1826-s001.docx]
